# Periparturient mortality in merino ewes in Australia: Incidence, impact and the path to mitigation strategies

**DOI:** 10.1111/avj.13430

**Published:** 2025-03-15

**Authors:** B Kirk, T Clune, E de Looff, J Jones, S Barber, D McGill, L Rowlands, S McGrath, J Kelly, A Whale, C Jacobson, E Glanville

**Affiliations:** ^1^ Melbourne Veterinary School, Faculty of Science University of Melbourne Melbourne Australia; ^2^ Murdoch University, Centre for Animal Production and Health, Food Futures Institute Murdoch University Murdoch Australia; ^3^ Pinion Advisory Dowsing Point Australia; ^4^ Central Queensland University Norman Gardens Australia; ^5^ Millicent Veterinary Clinic Millicent Australia; ^6^ Animal Health and Nutrition Consulting Coonamble Australia; ^7^ Livestock Logic, Apiam Animal Health Hamilton Australia; ^8^ Well Stock Consulting Bathurst Australia

**Keywords:** dystocia, lambing period, metabolic disease, sheep, survival

## Abstract

Managing breeding ewe mortality is a priority for the Australian sheep industry. The periparturient period carries the highest risk of mortality, but the incidence and causes in Merino ewes are not well characterised. Here, we outline the reported incidence of annual and periparturient mortality for Australian Merino ewes, causes and risk factors for ewe mortality and current recommendations for managing periparturient ewes and gaps in the literature. The mean incidence of periparturient mortality reported in Merino ewes ranges from 0.3% to 11.4%. However, there are challenges with reporting mortality figures in extensive production systems due to the nature of record keeping and farm characteristics. Studies reporting causes of periparturient mortality indicate that these are typically multifactorial, with metabolic disease and dystocia likely to be important. Identifying potential mitigation strategies has the potential to improve productivity, profitability and welfare on Australian farms.

Ewe mortality is a priority for sheep meat and wool industries worldwide due to potential impacts for animal welfare and production losses.^1^, ^2^, ^3^, ^4^ The Australian sheep industry consists of predominantly extensive production systems. This presents challenges in determining the incidence, risk factors and causes of both annual and periparturient ewe mortality.^1^ Ewe mortality ranging from 2% to 10% per annum has been reported in Australia,^5^, ^6^ with annual mortality of up to 25% reported in individual flocks.^6^ The periparturient period is a time of increased ewe mortality risk.^2^, ^4^, ^7^, ^8^, ^9^ However, breed‐specific incidence of periparturient ewe mortality and causes of death are not widely reported.^1^, ^9^

There are approximately 30 million Merino ewes in Australia, representing 64% of Australia's total ewe breeding flock.^10^ The Australian Merino encompasses a range of genotypes,^11^ which are managed in diverse production systems and environments, from high‐to low‐rainfall regions,^12^ resulting in different health and production challenges. Despite the importance of the Merino ewe to Australian sheep production, there has not been comprehensive, nation‐wide reporting of breed‐specific incidence of periparturient mortality, causes of death and risk factors.

Strategies that reduce periparturient Merino ewe mortality may benefit animal welfare and productivity directly via ewe production and indirectly via improved lamb survival. Australian guidelines for best practice management of ewes exist in extension programmes, but recommendations typically focus on improving lamb survival and often are not specific to genotype.^13^, ^14^, ^15^, ^16^ Determining the causes of and risk factors for periparturient mortality in Merino ewes will enable the inclusion of practical, cost‐effective and evidence‐based interventions in extension programmes targeting reduced mortality specific to genotype and production system. This review aims to describe the incidence of periparturient Merino ewe mortality in Australia, identify associated risk factors and describe the impacts for producers and industry.

## Ewe mortality and animal welfare in the Australian sheep industry

Animal welfare varies considerably within and between farms because of differences in management strategies, human intervention, intensity of the production system and environment.^17^ Most Australian sheep are farmed in extensive farming systems. Consumers often perceive extensive systems to achieve better animal welfare outcomes because animals have the freedom to exhibit natural behaviour in their natural habitat.^1^, ^18^ However, extensive production systems present their own challenges, including reduced opportunity for close monitoring of animals that may reduce opportunities to identify sick animals, diagnose and treat disease.^1^, ^19^ Furthermore, nutrition, climate and the presence of predators are highly variable in these natural environments.

Ewe mortality can be viewed from the perspective of both sheep producers and the broader community. The perspective of sheep producers on animal welfare is critical, as producers are best placed to identify and act upon welfare concerns on their farm. Further, the well‐being of producers is also linked to that of their stock.^1^ Consumer and community perceptions are important because these influence the industry's ‘social licence’^1^ Factors that may increase the emphasis placed on animal welfare in extensive systems in the future include increased societal awareness and social media.^20^ Livestock industries recognize the importance of demonstrating animal welfare outcomes to consumers and the broader community.^18^ The 2020 Meat Industry Strategic Plan calculated the downside risk to the industry related to negative welfare perception by the public to be well over AUD 3.5 billion to 2030 and the largest risk quantified in the report.^21^ Therefore, identifying risk factors for ewe mortality and implementing strategies that reduce mortality benefits the animals and producers, as well as improving societal perceptions.

## Defining ewe mortality

Mortality rate (the number of deaths in a population over time) is widely used as a measure of livestock health, productivity, and as an indicator of welfare.^22^ Measuring annual and periparturient mortality rates is the key starting point to determining the need for mitigation, identifying risk factors and ultimately reducing mortality rates. Part of the challenge in quantifying mortality rates is the difficulty in maintaining accurate mortality records in extensive farming systems with large flocks and paddocks that are large or poorly accessible. Mustering may occur relatively infrequently and may be incomplete.^23^ Carcases may also be difficult to find or consumed by predators. Theft of livestock may also cause discrepancies in stock tallies. Therefore, death is often assumed rather than confirmed.

Mortality records may be less commonly recorded in Merino flocks compared to meat‐breed flocks, with producers citing a lack of time as the most common reason for not keeping mortality records.^1^ Other reported barriers to recording ewe mortalities included that producers did not perceive value in the information or did not want to know.^1^ For sheep enterprises that do record mortality, accuracy remains a challenge. An Australian study reported that sheep mortalities (Merino and non‐Merino) were underestimated on around half of the farms studied, and only around a quarter to a third of mortality records agreed with stock tallies.^1^, ^24^ Similar challenges were reported where undetermined deaths were recorded with producer‐informed disease surveillance.^9^ Producers with better overall record keeping may also be more likely to record mortality data. These producers may also have better management and subsequently lower mortality than the industry average. Therefore, self‐reported data may be biased.^1^, ^24^

What constitutes a death, and thus underpins reported ewe mortality rates, varies between studies. Often mortality is defined by the absence of ewes at key events, such as pregnancy scanning, lamb marking, weaning or shearing^5^, ^16^, ^25^ or between two key events.^26^ Some studies included ewes that were expected to have died without intervention in calculating mortality.^7^ Therefore, the method used to determine ewe mortality rate must be considered when reviewing studies and reported rates.

The period for which mortality is calculated also varies between studies. The periparturient period is considered the period of highest risk for ewe mortality because the physiological events in late pregnancy and during parturition and early lactation increase the risk of death.^2^ However, there is inconsistency regarding when the periparturient period begins and ends. For example, McQuillan et al. defined this period as being between ewe allocation to lambing paddocks and lamb marking,^27^ whereas Pfeiffer defined the periparturient period as including the two months before lambing commenced through to three months after lambing.^9^

### Quantifying periparturient mortality rates in Merino ewes

Several studies have reported annual and periparturient mortality rates of ewes in Australia and New Zealand (Table 1 and Table 2). Anecdotally, Merinos have greater mortality rates in comparison to other breeds. However, there is limited literature reporting specifically Merino ewe mortality. Mean annual mortality in Merino ewes ranges between 3% and 11% with mortalities of up to 28% in individual flocks (Table 1).^32^ Mean periparturient mortality ranged from 0.3% to 11% (Table 2), with most studies reporting between 2 and 5% mortality rates, which is consistent with 2.0–2.5% periparturient mortality recently reported for non‐Merino ewes in Australia.^27^

**Table 1:** Summary of studies reporting annual Merino and non‐Merino ewe mortality in Australia

Author	Methodology	Sheep breed	n flocks or farms	n ewes	Location	Year	Ewe mortality		Cause of death determined^a^
							Average (%) (& time period, if not annual)	Range (%)	
Harris & Nowara (1995)^28^	Survey	Merino and crosses	227 flocks Twin‐bearing	135,686	VIC	1987–1989	Merino: 5.7 Non‐merino 10.9	5.1–13.6	Yes; flystrike, periparturient deaths (metabolic disease, other specific causes not defined), heliotrope poisoning
Trompf et al. (2011)^29^	Producer survey	Mixed	na	885,095	NSW, SA, TAS, VIC, WA	2008–2010	na	2.8–4.9	No
Kelly et al. (2014)^5^	Experiment and case study	Merino	6 farms	1440	NSW	2007–2009	10 (over the two years of the study)	6–22	No
Dever et al. (2017)^30^	Longitudinal study	Border Leicester X merino	5 farms	1200 twin‐bearing	NSW	2013–2014	6.5	6.3–6.7	No
Doyle (2018)^1^	Survey	Merino and non‐merino	32 farms	na	VIC	2017	4.7	0.2–14.4	No
Horton et al. (2018)^31^	Retrospective data analysis	Merino and crosses	8 flocks	4613	NSW, VIC, SA, WA	2007–2011	Flystruck: 7.1 Not flystruck: 4.3 (length of the trial; lambs born from 2007 to 2011)	na	No
Watt et al.^32^	Survey	Merino and non‐merino	225 farms	na	NSW	2010	5.4 (12 months)	0.1–28.6	Yes; periparturient deaths, foot abscess
Robertson & Friend^33^	Retrospective data analysis	Merino	1 flock ewes aged up to 2.5–9.5 years old	381	NSW	2006–2010	na	2.4–11.5	Yes; late pregnancy or lambing complications (specific causes not defined), Phalaris toxicity, ruminal acidosis
Australian wool Innovation^34^	Producer survey	Merino	1203 producers	na	NSW, VIC, WA, SA, TAS, QLD	2021	3	na	No

^**a**^ In studies where the cause of death was described, the three most commonly reported causes of ewe death are listed. The cause of death includes both producer‐reported classification and veterinary post‐mortem classification.

ITM, injectable trace mineral; na, not available, either not reported or unable to determine from the study; NSW, New South Wales; SA, South Australia; TAS, Tasmania; VIC, Victoria; WA, Western Australia.

**Table 2:** Summary of studies reporting periparturient (or part of year) Merino and non‐Merino ewe mortality in Australia

Author	Methodology	Sheep breed	n flocks or farms	n ewes	Location	Year	Ewe mortality		Cause of death determined^a^
							Average (%) (& time period)	Range (%)	
McGrath et al.^6^	Survey Ewes grazing on dual purpose wheat crops in the periparturient period	Mixed	43 farms	na	NSW	2005–2010	Case group (experienced losses that were abnormally high): 8.6; control group (ewe mortality ‘normal’): 1.8 (‘late pregnancy and lambing’)	Up to 25	Yes; metabolic disease, dystocia, foot abscess
Munoz et al.^17^	Longitudinal study	Merino	1 flock	100	VIC	2015	5 (mid pregnancy to weaning)	na	No
McQuillan et al.^35^	Observational study	Maternal	80 flocks	235,111	WA, SA, VIC, NSW	2019 2020	2.5 (2019) 2.0 (2020) (set stocking to lamb marking)	0.5–5.9 (2019) 0.8–4.7 (2020)	Yes; septicaemia, primary dystocia, trauma
Clune et al.^36^	Cross‐sectional study	Merino hoggets Non‐Merino ewe lambs	11 flocks 18 flocks	2170 3858	WA, SA, VIC	2018–2020	0.3 0.7 (scanning to lamb marking)	na	No
Hutchison et al.^25^	Producer survey	Merino + non‐merino	79 farms	413,702	NSW, SA, TAS, VIC, WA	2018–2020	Maiden merino 1.7 versus adults 2.4 Maiden non‐merino 2.6 Adults 2.8 (scanning to lamb marking)	na	No
Gonzalez‐Rivas et al.^37^	Experimental study comparing use of trace mineral supplement (ITM) versus control	Mixed	5 farms	1484	VIC	2018–2019	ITM group (1.17) control group (2.49) (na; trial ran from 30 days before joining, to weaning)	na	No
Thompson et al.^38^	Producer survey	Merino + non‐merino	105 flocks	na	NSW, SA, TAS, VIC, WA	2017–2018	Merino ewe mortality: Single 1.3, twin 2.5, triplet 6.7 Non‐merino Single 1.5, twin 3.1, triplet 4.9 no sig. Difference between breeds (pregnancy scanning to lamb marking)	na	No
Lockwood et al.^16^	Experimental study comparing ‘high’ and ‘low’ mob size in triplet‐bearing ewes	Merino + non‐merino	12 farms, 31 ‘high’ mobs, 47 ‘low’ mobs	‘High’ group average 63/mob ‘Low’ groups average 20/mob	NSW, VIC, WA	2019–2021	High mob size: 6.3 Low mob size: 5.1 (from average of 135 days from joining start, through to lamb marking)	na	No
Haslin et al.^26^	Experimental study comparing ‘high’ and ‘low’ BCS in triplet‐bearing ewes	Merino + non‐merino	19 farms: 12 maternal, 7 merino	Maternal: High (1135), low (1019) Merino: High (545) Low (551)	NSW, SA, VIC, WA	2019–2021	Maternal: High 6.1, low 4.8 Merino: High BCS 4.2 Low BCS 11.4 (pregnancy scanning to lamb marking)	na	No

BCS, body condition score; ITM, injectable trace mineral; na, not available, either not reported or unable to determine from the study; NSW, New South Wales; SA, South Australia; TAS, Tasmania; VIC, Victoria; WA, Western Australia.

^**a**^ In studies where the cause of death was described, the three most commonly reported causes of ewe death are listed. The cause of death includes both producer‐reported classification and veterinary post‐mortem classification.

There are biases and gaps in the literature that mean further investigation of mortality rates in different regions is warranted to inform how periparturient Merino ewe mortality can be managed. Large mortality events may be more likely to be investigated and reported and may be related to extreme seasonal conditions or disease outbreaks.^6^, ^28^, ^32^ Most studies reported limitations and challenges determining periparturient mortality rates such as being confined to one geographic area, not being specific to Merinos or reporting on annual (not periparturient) mortality. Mortality and the endemic diseases associated with sheep mortality are highly variable across seasons, production systems, sheep genotypes and geographical areas.

### Determination of causes for periparturient mortality

Most studies reporting ewe mortality do not report the cause of death, and in those that do, the cause of death is mostly ‘producer‐reported’ rather than a diagnosis made via veterinary post‐mortem (Tables 1 and 2). This introduces error, as many conditions cannot be diagnosed or are underreported by external examination alone. A recent study of mortality in maternal (non‐Merino) ewes reported a greater proportion of ewes with an ‘unknown’ cause of death from producer records compared to the subset of cases where veterinary post‐mortem examinations were used to identify the cause of death.^27^ Only about 10%–15% of mortalities reported in the literature include veterinary involvement.^9^, ^28^

## Costs associated with ewe mortality

The loss of a periparturient Merino ewe is of financial impact for the sheep producer because of the loss of both capital (the ewe herself) and expected return for the investment (lamb and fleece).^2^ There is also the foregone income from selling excess young sheep if more lambs must be retained as replacements or increased costs through purchasing more ewes to retain breeding flock numbers. Bioeconomic modelling on the impact of sheep mortality on farm income using Model of an Integrated Dryland Agricultural System indicated that costs of ewe and lamb mortality are more sensitive to the sale price of sheep (i.e. meat value lamb and the ewe) than the price of wool.^39^

A number of studies have quantified the farm‐level and industry cost of Merino ewe mortality. At the industry level, bioeconomic modelling indicated that increasing the survival of ewes at lambing had the third highest potential payoff for the industry, behind increasing the survival of twin‐born lambs and increasing reproduction from ewe lambs.^40^ At the farm level, a cost–benefit analysis using 10‐year median prices for a self‐replacing (but not breed‐specific) flock reported that reducing ewe mortality from 4.9% to 3.9% would give a benefit of AUD 403 per 1000 DSE (dry sheep equivalents).^1^ Shephard et al estimated the cost of dystocia alone to be AUD 528.7 M across the Australian flock of 68 million sheep, but this value included both ewe and lamb mortalities and was not specific to Merinos.^41^ Most of the financial loss associated with dystocia is attributable to lamb mortality, but ewe mortalities associated with dystocia have been estimated to reduce national farm income by AUD 81 M and cause 297,500 ewe deaths per annum across a breeding flock with 34 million ewes mated.^39^ The costs from other causes of periparturient mortality across all sheep breeds have been estimated, including mastitis (AUD 104.1 M), hypocalcaemia (AUD 44.4 M) and pregnancy toxaemia (AUD 30.3 M).^41^

The ‘Fit To Join’ tool involved producers assessing ewes' condition score (CS), age, teeth, feet and udder health.^14^ These key assessment criteria for both Merino and non‐Merino ewes were used before ewes being joined, to improve productivity and welfare outcomes. It was found that ‘unfit’ ewes (which did not pass the examination criteria) were three times more likely to die than ‘fit’ ewes. The economic benefit for classing and subsequent culling of ewes unfit to join was estimated to be between AUD 4 and AUD 8 per ewe.^14^

## Factors implicated in ewe mortality

### Environment

‘Environment’ includes ambient temperature, topography, soil type, rainfall, wind speed, and pasture species, which in turn influence chill index, risk of flystrike and gastrointestinal nematode burden, and therefore the risk of associated illness and death.^32^, ^42^ Environmental factors may explain variation in mortality between years for a specific property, as well as variation between locations and production systems.

Doughty et al. (2019) reported environment (including location and year) was an important factor affecting ewe mortality in Merino flocks being managed according to a ‘standard best practice protocol’.^22^ Seasonal variations can also impact mortality rate and cause of death, including through impacts on risk factors for specific conditions including foot abscess and gastrointestinal parasitism.^5^, ^32^

### Ewe body weight and body CS

Most studies assessing ewe bodyweight and CS focus on the implications for birthweight and survival of lambs. However, ewe liveweight and CS also have implications for diseases associated with periparturient ewe mortality, including dystocia and metabolic diseases.^43^

Low or decreasing liveweight or CS have been associated with an increased risk for ewe mortality.^4^, ^5^, ^22^, ^44^ The risk of mortality for triplet‐bearing Merino ewes between pregnancy scanning and lamb marking was increased when ewes were managed to have lower CS (2.8) compared with ewes managed to have higher CS (3.0) at lambing.^26^ The association between ewe body CS during pregnancy and dystocia is inconsistent, and the optimal CS to reduce the risk of ewe mortality is not well defined for specific pregnancy types (single or multiple‐bearing ewes), ewe genotype and production system.^3^ Apart from dystocia, an increased risk for the development of pregnancy toxaemia, metritis or mastitis was reported in Greek dairy ewes when ewe CS was outside the range of 2.75 to 3.5.^45^ It is likely that liveweight and CS parameters have different risk thresholds for specific causes of ewe death. Further investigation to inform recommendations that are specific to genotype (breed) and consider interaction with other risk factors including age, litter size (single, twin or higher order multiples) and production system (intensive vs. extensive systems) is warranted.

### Ewe age and parity

Ewe age and parity influence periparturient mortality, but these factors are confounded with litter size as older ewes are more fecund.^3^ Lower mortality was reported for maiden (primiparous) Merino ewes lambing at two years of age compared with mature multiparous ewes on the same farms.^25^ Higher risk for dystocia was reported for ewes three years old or older and particularly multiple‐bearing ewes for both Merinos and non‐Merinos across eight Information Nucleus Flocks across Australia.^46^ Other studies have reported no effect of age group on mortality in Merino ewes.^5^ Non‐Merino ewes over 5 years old at lambing are at increased risk of periparturient mortality,^27^ but a recent study showed no increase in mortality of Merino ewes as they aged between 5.5 and 9.5 years old, though this study was only on one relatively small flock of around 380 ewes.^33^

### Genetics and heritability of mortality

There are limited reports on the heritability of survival in Merino ewes with reported links between sire and ewe survival^22^ as well as weaner and lamb survival.^47^, ^48^ Heritability of 0.06 for survival in ewes from birth to 4 years of age has been reported from the Merino Lifetime Productivity project.^49^ Survival was found to be moderately negatively correlated with wrinkle and breech cover, indicating that plain‐bodied ewes with less breech cover were more likely to survive.^49^ Low heritability means that selection for survival is expected to result in small improvements over time. Consequently, implementing management strategies to address mortality remains a high priority. However, genetic gains made through selection are cumulative and permanent, so this should be considered as part of an overall suite of recommendations focused on ewe survival.

## Ewe mortality in the periparturient period

Mortality in the periparturient period has been classified into pre‐partum (metabolic disorders, infectious sequelae to abortion, clostridial infections); during lambing (obstetrical problems, trauma and haemorrhage); and post‐partum (septicaemia‐toxaemia due to acute metritis or mastitis, metabolic disorders or clostridial infections).^2^ Some disease processes are not directly related to pregnancy and lambing, but periparturient ewes may be more susceptible due to immunosuppression or other factors related to pregnancy, lactation or management. These include pneumonia,^2^ gastrointestinal parasitism,^50^ other infectious agents such as *Salmonella*, metabolic acidosis (occurring due to grain supplementation to meet energy requirements) and Ovine Johne's Disease. Overall, the incidence and risk factors for these diseases in Australian Merino ewe flocks during the periparturient period are not well described.

## Pre‐partum causes of mortality

### Pregnancy toxaemia

Pregnancy toxaemia is an important cause of ewe mortality on some farms in some years, with an estimated 80% mortality rate for clinical cases.^41^ The incidence of pregnancy toxaemia and its contribution to periparturient Merino ewe mortality remain poorly described, with a wide range of reported incidence in Merino and non‐Merino flocks (1% to 12%).^28^, ^35^, ^51^ Further investigation is warranted to understand the importance of this disease for Merino ewes.

Pregnancy toxaemia occurs when the glucose requirements of the pregnant ewe are unable to be met by her feed intake and glycogenic stores, resulting in a negative energy balance.^43^ Ewes in the last 4 weeks of gestation are particularly susceptible. Hypocalcaemia and hypomagnesaemia may also develop secondary to inappetence.^52^ The challenges associated with differentiating metabolic diseases to determine aetiology should be considered when comparing studies reporting causes of death.^9^, ^32^ Ewes that recover from pregnancy toxaemia have an increased risk of dystocia when lambing.^52^ Risk factors for pregnancy toxaemia include multiple foetuses; feed intake that does not meet nutritional demands; sudden reduction in intake; very high or very low CS; and comorbidities that reduce grazing (e.g. lameness).^2^, ^43^

### Hypocalcaemia

The incidence of hypocalcaemia in Merino ewes is not well quantified despite anecdotal reports of widespread occurrence. Shephard et al. estimated that 0.2% to 0.4% of ewes are affected depending on rainfall zone and class of sheep, but this was based on limited data.^41^ Hypocalcaemia was the third most commonly diagnosed cause of death in one study on non‐Merino ewes during the periparturient period and was associated with most cases investigated on some farms where post‐mortems were performed.^35^

Hypocalcaemia in ewes usually occurs in the last month before lambing but has also been reported less commonly in younger or non‐pregnant animals.^43^ The presentation differs from that of pregnancy toxaemia, and the progression of this disease is much faster, with death occurring in 1–2 days. Affected animals are alert but ataxic to recumbent, remain aware of their surroundings, are unable to rise, and muscle tone is decreased. A mortality rate of 40% was estimated for hypocalcaemia cases that are left untreated.^41^

Subclinical hypocalcaemia may increase the risk of obstetrical problems such as prolonged parturition and dystocia, prolapse and retained foetal membranes.^3^, ^53^ Subclinical hypocalcaemia has been implicated in uterine prolapse and retained placenta in cattle, but it is unknown if this is also the case in ewes.^3^ Administration of calcium was not protective against vaginal prolapse,^54^ though it has been reported as a risk factor in other studies.^55^

Risk factors for hypocalcaemia include older age and pregnancy with multiple foetuses. Older ewes are less efficient at resorbing calcium from bone, and bone calcium reserves may be depleted by successive pregnancies and lactations. Multiple foetuses impose higher calcium demands on the ewe than single foetuses. Hypocalcaemia may be precipitated by time off feed due to yarding, inclement weather precluding grazing, or mustering activities. Nutritional risk factors may include grain feeding (including long periods of grain feeding with reduced access to green feed), grazing green cereal crops (such as wheat, oats or barley)^56^ or grazing stubbles due to either low level of calcium, low calcium: phosphorus ratio, high potassium: sodium ratio and / or high dietary anion cation difference. A change from short dry feed to lush green feed in mid to late pregnancy is also a risk factor for hypocalcaemia due to the lower calcium concentration in green feed.^57^ The grazing of vegetative cereal crops has become more popular in areas of Australia where both crops and livestock are produced^56^, ^58^ and may increase the risk of hypocalcaemia.^6^ Mineral supplementation can improve calcium status of ewes.^59^ However, a recent study found calcium supplementation of periparturient ewes did not increase ewe survival.^60^ Feeding diets with low dietary anion cation difference may reduce parturition time in twin‐bearing Merino ewes,^61^ but reducing dietary anion cation difference through supplementation for ewes grazing pasture or crops is challenging.^62^ Further work is required to define requirements for mineral supplementation, optimum methods for mineral supplement delivery and the effect of supplementation on the survival of Merino ewes and their offspring under different conditions.

### Vaginal or cervico‐vaginal prolapse

Vaginal, or cervico‐vaginal prolapses (referred to hereafter as vaginal prolapses), occur before lambing. If diagnosed and treated early, the ewe may successfully undergo parturition, though the condition may recur in subsequent pregnancies.^55^ Reported risk factors include ‘overfatness’ ewes pregnant with twins or higher order multiples, particularly older ewes, ewes with ‘short docked’ tails, and ewes run in hilly terrain during late pregnancy.^55^ The definitive cause of vaginal prolapse is not known; therefore, prevention is based on the reduction of risk factors. One preliminary study in New Zealand found that the administration of Vitamin A, D and E during pregnancy reduced the incidence of vaginal prolapse in sheep.^63^

The incidence of vaginal prolapse in Australian Merino ewes is not well reported. In non‐Merino ewes, vaginal prolapse was diagnosed in 1% (in 2019) and 7% (in 2020) of deaths investigated by veterinarians.^35^ A study using Romney and composite ewes in New Zealand reported vaginal prolapse for only 23 of the 1003 reported ewe deaths.^64^ Although vaginal prolapse is unlikely to be a major contributor to Merino ewe mortality at the industry level, it may be significant on some farms in some years. Potential sequelae of vaginal prolapse include dystocia; trauma or infection to the externalised tissue; dorsal vaginal wall rupture; or prolapse of other organs, all of which may result in death or necessitate euthanasia of the ewe.

## Causes of mortality that occur during parturition

### Dystocia

Dystocia is the term for a difficult and prolonged birth.^43^ Dystocia can be classified as maternal dystocia (originating from the ewe) or foetal dystocia (originating from the lamb).^3^ Resulting trauma, either due to the dystocia itself or from assistance provided during lambing, may result in sequelae including haemorrhage, intestinal herniation, uterus prolapse and infection, all of which can be fatal to the ewe.^65^

Despite its frequent occurrence, little is known of the risk factors for dystocia in Merino ewes. Studies that do report the rates of dystocia show a variable incidence.^39^ Dystocia and its sequalae are the main cause of death in non‐Merino ewes during the periparturient period in southern Australia, with dystocia identified in 29%–41% of cases where a post‐mortem was performed by a veterinarian. Dystocia was also the most important cause of death reported by farmers in the same study, although dystocia is likely underreported by farmers as veterinary post‐mortems demonstrated that only two thirds of dystocia cases could be identified using only external examination.^27^ Producers reported dystocia as the cause of ewe death in 18% of the recorded dead ewes that were 3 years and older and in 6% of ewes that were rising 1 year old for Merino and non‐Merino flocks from the Mallee region of Victoria in which overall ewe losses exceeded 5% per annum.^28^

### Risk factors for dystocia

Factors associated with the risk of ewe mortality due to dystocia include ewe parity, litter size,^27^, ^66^ lambing year;^22^, ^66^, ^67^ ewe breed and month of lambing;^66^ and age at first lambing.^35^ However, many of these studies included non‐Merino ewes or did not separate ewes by breed in the analysis. Litter size was reported as a significant risk factor for dystocia in Australian Merino ewes, with triplet births found to be associated with more dystocia than twins, and high birthweight found to be associated with dystocia in single births.^46^

Reported genetic correlations between reproduction traits (including dystocia) had large standard errors, due in part to the relatively low number of post‐mortem results available and because the data were binary.^68^ Lambing Ease score had the highest genetic correlation of potential indicator traits^68^ and Australian Sheep Breeding Values (ASBVs) now include Lambing Ease (Direct) and Lambing Ease (Daughters). The accuracy of the Lambing Ease ASBV is reduced when births are not actually observed, and it is difficult for studs and seedstock breeders to collect the data required for breeding values.^69^

#### Lamb birthweight

Analysis of Merino and crossbred flocks found that lambs with a birthweight outside the range of 4.5‐5 kg had an increased risk of dystocia.^46^, ^68^ Most of the studies describing the relationship between lamb birthweight and dystocia have focused on lamb rather than ewe survival and are based on lamb post‐mortem and survival data^46^, ^68^, ^70^, ^71^ or Lambing Ease score.^68^

Lamb birthweight is influenced by genotype (ewe and sire), litter size (single, twin or higher order multiple) and ewe nutrition during pregnancy.^3^, ^68^ Birthweight and lambing ease ASBVs may be used to reduce the risk of foetal‐pelvic disproportion dystocia in single‐born lambs with high birth weights.^68^ However, as with the Lambing Ease ASBVs, data required to inform lamb birthweight ASBVs are challenging to collect under extensive conditions, and lamb birthweight ASBVs are currently not widely available for Merino rams. Therefore, while genetics and management of Merino ewes will go some way to reducing the incidence of dystocia, the complex interplay of dystocia type, genotype, litter size and production systems means that further work is needed to provide appropriate recommendations to reduce the risk of dystocia.

#### Grazing oestrogenic pastures

Moule (1961) reported that ewes grazing oestrogenic clover (*Trifolium* spp.) had rates of dystocia of 30%–40%, with 15%–20% mortality of all ewes mated.^72^ The dystocia is thought to be due to either primary uterine inertia or failure of the cervix or vulva to dilate.^3^ Common varieties of oestrogenic clover include Dwalganup, Geraldton, Dinninup and Yarloop.^73^ In 2002, it was estimated that 10–15 million ewes in Australia were affected by oestrogenic clovers.^74^ In a 2019 survey, 60% of clover samples had concentrations of formononetin that exceeded the safe level, though the authors noted potential bias as sheep producers who were concerned about their pastures were more likely to return the sample kits provided.^75^

#### Previous dystocia

The risk of dystocia in subsequent lambings is specific to the type of dystocia experienced (high or low birthweight) and is strongly related to litter size.^46^ There may be an opportunity to identify high‐risk ewes by history and age to inform subsequent joining decisions (depending on availability of rams with appropriate ASBV for birthweight, Lambing Ease [Direct]), and additional monitoring during pregnancy and lambing.

### Iatrogenic trauma during assistance with lambing

The incidence of iatrogenic trauma secondary to dystocia or prolapse in Merino ewes has not been widely reported. This can be reduced by managing risk factors that predispose ewes to conditions where obstetric intervention is required, training staff to prevent excessive force from being used if assisting ewes during lambing, and maintaining hygiene as much as possible during lambing.^65^

## Common post‐partum causes of mortality

### Hypomagnesaemia

Clinical hypomagnesaemia is reported to be relatively uncommon in Australian sheep but may cause ewe deaths on some farms in some years.^27^, ^43^ Hypomagnesaemia is rarely identified in ovine submissions to Australian veterinary diagnostic laboratories without current hypocalcaemia.^76^ For example, of approximately 12,200 diagnostic ovine submissions submitted to the Elizabeth Macarthur Agricultural Institute (New South Wales Department of Primary Industries) between July 2006 and July 2021, only 16 cases included sheep with serum magnesium concentrations of less than 0.50 mmol/L, and only 7 cases included sheep with aqueous humour magnesium concentrations of less than 0.48 mmol/L.^77^ However, sporadic events with significant ewe mortality in north‐east Victoria have been reported.^78^ The highest risk period is during the first 8 weeks of lactation and in ewes grazing rapidly growing grass‐dominant pastures or cereal crops with high potassium, low sodium, and high crude protein. Hypomagnesaemia causes tetanic convulsions, though ewes may be found dead because of the very rapid progression of the disease.

### Mastitis

The incidence of mastitis in Australian Merino flocks has not been extensively studied but can be a significant problem on some farms in some years.^79^ Clinical and sub‐clinical mastitis was estimated to cost around AUD 104.1 M in 2022.^41^ Adverse climatic conditions and high stocking rates have been associated with clinical mastitis incidences of greater than 10% with subsequent ewe mortality and culling.^79^ The prevalence of subclinical mastitis in the Armidale region of New South Wales was reported to be around 14%.^80^

Common aetiological agents of clinical mastitis in Australia include *Staphylococcus aureus* and *Mannheimia haemolytica*.^43^ Mastitis can cause ewe deaths in the periparturient period. Ewes may present with clinical signs of mastitis. However, ewes may simply be found dead if there is limited opportunity to observe ewes closely, particularly when the bacterial strains present result in acute or sub‐acute disease. In surviving ewes, mastitis may cause lamb mortality, reduced lamb growth rates due to decreased milk production or subsequent culling of ewes due to udder damage. Risk factors for mastitis include milk stasis (for example, if lambs do not suckle effectively or die); teat or udder damage (including from prior lactations^2^ or at shearing); or environmental contamination.^46^

### Metritis

Metritis risk is increased after abortion, stillbirth, dystocia, vaginal or uterine prolapse, retained placenta or systemic disease.^65^ Generally, if ewes are identified and treated in a timely manner, they recover quickly, and future reproduction is not affected.^2^, ^65^ However, clostridial infections may result in toxaemia and rapid death of ewes.^3^, ^65^ The risk of abortion and stillbirth is higher in younger ewes that are more susceptible to infectious diseases, and especially ewes lambing at 1–2 years old.^36^, ^81^, ^82^

### Uterine prolapse

Although the incidence of uterine prolapse in Merino ewes is not well reported, uterine prolapse is not common in Australian ewes. Uterine prolapse was reported in 5%–10% of cases for non‐Merino ewes submitted for post‐mortem.^27^ Only one farm out of the 71 surveyed reported uterine prolapse (with 0.5% of ewes on that farm affected) in a study on Merino ewes grazing wheat in New South Wales.^58^

Uterine prolapse may be a sequelae to dystocia, especially after prolonged straining or vaginal damage, or due to oestrogenic clovers.^43^, ^72^ There is also some evidence that hypocalcaemia is implicated in the incidence of uterine prolapse.^65^ The prognosis for ewe survival is relatively good if the condition is treated promptly, and the uterus is not damaged,^43^ although identifying and treating ewes without disturbing other lambing ewes presents a challenge in many extensive production systems.

### Concurrent conditions: Parasitism

Flystrike and gastrointestinal parasitism may be implicated in periparturient ewe mortality. Gastrointestinal parasitism is not exclusively a periparturient disease, but periparturient relaxation of immunity increases ewe susceptibility to nematodiasis.^50^ ^,^ ^83^ Merino ewes with a worm egg count (WEC) of greater than 1200 eggs per gram caused by *Haemonchus contortus* had a 3.6 times greater risk of mortality than ewes with low WECs.^5^ High burdens of *Teladorsagia* and *Trichostrongylus* spp. can also lead to clinical parasitism or death of ewes.^50^ ^,^ ^84^

Merino ewes may also be affected by external parasites in the periparturient period. In a study assessing the impact of flystrike in a non‐mulesed Australian Merino flock, researchers reported the incidence of flystrike at 17% with 2% of struck sheep dead within 7 days of detection.^31^ This was consistent with another study where 1.8% of Merino ewes that were affected by flystrike died within a week.^22^ High worm burdens or hypersensitivity scouring can predispose ewes to breech strike.^85^

### Concurrent conditions: Foot health

Diseases causing lameness, such as foot abscess and footrot, may contribute to ewe mortality during the periparturient period.^32^ Reduced feed intake because of lameness increases the risk of pregnancy toxaemia and hypocalcaemia in pregnant ewes.^43^, ^86^

Risk factors for foot abscess in sheep include high rainfall and lush pasture with more than 30% clover.^32^, ^86^ High bodyweight, including heavily pregnant ewes, is also a risk factor.^41^ Pregnancy toxaemia is a common complication of foot abscess reported by producers.^6^, ^86^ Foot abscessation detection rates ranged from 0.03% to 28% in central NSW flocks.^86^ Seasonal variation can impact foot health and impacts ewe mortality, with 20% of producers in New South Wales reporting foot abscess as the most common cause of ewe mortality (alone or in association with hypocalcaemia or pregnancy toxaemia) during a wetter‐thanthan‐average year and a further 30% of producers indicated that foot abscess was a problem on their properties.^32^ Another NSW survey reported ewe mortalities associated with foot abscess in 10%–26% of study groups where the estimated mortality rate due to foot abscesses ranged from 1.6 to 4.6%.^6^

Footrot is a contagious bacterial disease caused by *Dichelobacter nodosus* in association with other bacteria. The clinical expression of footrot is influenced by strain virulence, host susceptibility, and environmental factors.^87^ Estimates of the proportion of flocks affected by virulent footrot vary between states and range from 1 to 25%.^41^ To the best of our knowledge, there are no reports detailing ewe mortalities associated with footrot in Australia. In a trial conducted on wethers, infected with footrot in NSW from 1985 to 87, mortalities were higher in the group without preventative treatment (8% and 2% in the 2 years of the trial, respectively) than in the group that was foot bathed regularly (6% and 2%; not significant).^88^ Many of these deaths were due to flystrike. Further research is required to determine the contribution of footrot to ewe mortality across Australia.

### Concurrent conditions: Ovine Johne's disease (OJD)

Although not directly related to pregnancy or parturition, OJD may be implicated in periparturient ewe deaths. OJD can be a major contributor to mortality on farms where OJD is present and uncontrolled. For example, OJD was found to be the primary cause of death for nearly 70% of sheep mortalities (not just ewes) that underwent post‐mortem examination on 12 NSW farms with endemic OJD in 2002, and 75% of sheep mortalities that underwent post‐mortem on one farm in 2003.^89^

## Current recommendations for managing ewes to reduce periparturient mortality

### Lifetime ewe management (LTEM) principles

The LTEM programme is based on the differential management of single‐ and multiple‐bearing ewes from day 72 of pregnancy (around scanning) and feeding ewes to meet CS targets. Although the main focus of the programme is the improved survival and performance of the progeny, preferential feeding has benefits for the ewes, particularly for twin‐bearing ewes.

The LTEM programme provides a useful framework to introduce and establish practice change related to ewe mortality. Twin‐bearing ewes with higher CS fed to meet targets had higher survival (98% survival) compared with those in the control group (82% survival). Ewe survival was also higher in the single‐bearing ewes managed to meet targets (98% survival) compared with controls (95% survival).^90^ LTEM participants reported a 43% reduction in ewe mortality, from an average of 4.9% before completing the programme to 2.8% after completion.^29^

### Shepherding during lambing

Flock managers may monitor (‘shepherd’) their ewes during lambing. Human presence during labour, such as during traditional shepherding, can delay the process of parturition, which in turn can lead to dystocia and interfere with the ewe‐lamb bond.^19^ The review found no clear evidence that shepherding during lambing is helpful overall for the survival of either ewes or their lambs.^19^ However, monitoring ewes during lambing can result in more rapid identification of ewes requiring euthanasia, resulting in improved welfare outcomes for affected ewes.

Several reviews discuss the selection of ‘easy care’ sheep which do not require a lot of intervention even during lambing.^91^, ^92^ Although this has been mostly applied in the context of high input systems in the United Kingdom, the principle of selection for ‘easy care’ sheep can also be applied to Australian extensive systems with the opportunity to align sheep genotype and management strategies to the farm production system.^91^

### Pathways to adoption of practice change

Historically, adoption of established best practice on farms has been difficult to achieve due to factors including, but not limited to, farm‐specific, geographical, labour and external factors^1^ ^,^ ^29^ Other barriers to adoption included insufficient focus on reproduction within extension programmes; little perceived advantage from taking up the recommendations, and insufficient engagement by producers.^1^, ^29^

The LTEM extension programme for Merinos has been successful in instituting practice change in the Australian sheep industry.^29^ This programme is based on ‘experiential learning’ where participants practice new skills on several properties, including their own, and are presented with information both verbally and visually, originally over 2 years.^29^ A large proportion of surveyed participants adopted the management practices in the programme.^93^ Participants reported improvement in farm enterprise metrics, including increased stocking rate and lower ewe mortality.^93^ In a wider survey, 90% of producers who had participated in LTEM had changed their practices due to their involvement, compared with 12% of producers in the total survey population.^29^ Producers' perceived skill level in these management practices also increased, and 72% of participants indicated that they were more willing to undertake other training.^29^

The industry extension programme Making More from Sheep (MMFS) engaged over 20,000 participants between 2008 and 2016. Over half of the participants that were interviewed reported making changes on their farms after attending an MMFS programme.^94^

Flock‐level management and recording systems have been shown to be effective and valuable in reducing losses both in the short term and for successive seasons.^1^, ^9^, ^24^, ^27^, ^35^ Studies using producer interviews reported that best management practices were perceived as not too difficult to perform, and presenting accurate ewe mortality data was an important first step in the engagement of producers that did not currently assess ewe CS and nutrition.^1^, ^24^ Producers were more likely to carry out ‘best practice’ husbandry practices if they perceived these activities to be valuable.^24^ The path to reducing mortality rates in the Australian Merino ewe flock begins with recording farm mortality data and comparing farm data to ‘best practice’ mortality benchmarks. Achievable and cost‐effective mitigation strategies can then be presented, and producers supported to institute these management recommendations. However, to achieve this, producers must be motivated and engaged to overcome initial barriers to the adoption of these recommendations.

## Gaps in knowledge

Reporting periparturient mortality rates and causes of periparturient ewe mortality is hindered by challenges monitoring stock during lambing and the generally low level of veterinary involvement in investigating mortalities. This is reflected in the mortality rates reported for Australian Merino ewes and the lack of evidence quantifying risk factors for mortality in Merino ewes. Further, studies of Merino mortality are usually region‐specific. An approach at a national level would allow better understanding of periparturient ewe mortality and inform recommendations accounting for different production systems, management approaches and environments across Australia.

Furthermore, establishing the relationship between genetics and the management of risk factors for Merino ewe mortality would help guide targeted mitigation strategies.

Potential management changes that may reduce mortality, barriers to adoption and the complexities and nuances of instituting management changes on farm warrant further investigation to ensure future farm engagement programmes provide practical pathways to adoption. Economic impacts have been modelled in several recent studies, but the underlying assumptions used for cost–benefit analyses would be improved with better data for Merino ewe mortality in different production systems. Understanding the costs and efficacy of mitigation strategies for Merino ewe mortality can inform the value proposition for practice change and the adoption of best management recommendations that influence the survival of Merino ewes.

## Conclusion

Managing periparturient mortality in Merino ewes remains a priority for the Australian sheep industry. Ewe mortality impacts animal welfare and farm income through costs incurred and foregone income from meat and wool, as well as producer well‐being. The incidence of periparturient Merino ewe mortality has been reported, but limitations exist with regard to the variable methodology for the reporting of mortalities and the limited geographical areas of focus.

Although some studies have reported causes of death in Merino ewes, in most cases, diagnoses were not based on veterinary post‐mortems. Studies over a wider geographical area, including detailed record keeping of deaths as well as post‐mortems, would help identify the relationship between potential risk factors and mortalities, support economic modelling and inform sheep management recommendations that can improve ewe survival in Australian sheep production systems. This may enable cost‐effective, locally relevant mitigation strategies to be developed, with the aim of improving animal welfare and economic gains at both a farm and an industry level.

## Conflicts of interest and sources of funding

The authors have no conflicts of interest to declare.

## Data Availability

Data sharing not applicable to this article as no datasets were generated or analysed during the current study.
